# The Value of Antiseptics

**Published:** 1883-02

**Authors:** 


### The Value of Antiseptics.

In the course of the Presidential address at the meeting of the Yorkshire branch of the British Medical Association, (*British Medical Journal*) Dr. T. R. Jessop said:

As an instance of the extent to which, with antiseptic precautions, we may pursue a cancerous infiltration, I may mention the case of a hospital patient from whom I had previously removed the scirrhous mamma, and who had now returned with such a fulness between the clavicle and the deeply indented scar as to lead me to explore for secondary disease. On opening up the axillary and sub-pectoral spaces, I found them to be literally filled with adherent and infiltrating masses of growth, as is frequently the case, out of all proportion to the pre-existing signs.

For their extirpation prolonged tearing and dissection were needed, during the course of which, first of all, the pleural cavity was distinctly laid open, and subsequently the axillary artery, which had been imbedded, and, perhaps, displaced, was completely torn across, and yet, with all this, the woman was able to leave the hospital within three weeks, her wound soundly healed, and herself none the worse, apparently for the dangers through which she had passed. And again, as illustrating the advantages to be gained by perseverance in the removal of recurrent growths, I may select the case of a lady—a patient under the joint care of Mr. Halliday, of this town, and myself—who, after having during twelve consecutive months undergone no fewer than six separate operations (including the original amputation of the breast), for the removal of cancerous nodules affecting the cicatrix, the surrounding skin and subjacent tissues and the axillary lymphatics, passed upwards of two years of usefulness, in the enjoyment of good health, now again exerting herself beyond the average of women; and then died, without any sign of outward disease, from an acute thoracic affection of short duration.

